# Introduction and Development of Surface-Enhanced Raman Scattering (SERS) Substrates: A Review

**DOI:** 10.3390/nano14201648

**Published:** 2024-10-14

**Authors:** Jianping Peng, Yutao Song, Yue Lin, Zhenkai Huang

**Affiliations:** 1 School of Environment and Chemical Engineering, Foshan University, Foshan 528000, China; 15535522031@163.com (Y.S.); lly009612@163.com (Y.L.); 2 School of Materials and Energy, Foshan University, Foshan 528000, China

**Keywords:** SERS, substrates, preparation

## Abstract

Since its discovery, the phenomenon of Surface Enhanced Raman Scattering (SERS) has gradually become an important tool for analyzing the composition and structure of substances. As a trace technique that can efficiently and nondestructively detect single molecules, the application of SERS has expanded from environmental and materials science to biomedical fields. In the past decade or so, the explosive development of nanotechnology and nanomaterials has further boosted the research of SERS technology, as nanomaterial-based SERS substrates have shown good signal enhancement properties. So far, it is widely recognized that the morphology, size, composition, and stacking mode of nanomaterials have a very great influence on the strength of the substrate SERS effect. Herein, an overview of methods for the preparation of surface-enhanced Raman scattering (SERS) substrates is provided. Specifically, this review describes a variety of common SERS substrate preparation methods and explores the potential and promise of these methods for applications in chemical analysis and biomedical fields. By detailing the influence of different nanomaterials (e.g., metallic nanoparticles, nanowires, and nanostars) and their structural features on the SERS effect, this article aims to provide a comprehensive understanding of SERS substrate preparation techniques.

## 1. Introduction

One of the key points to realize the application of SERS enhancement technology is the preparation of SERS substrates [1,2,3,4,5]. Since the SERS phenomenon on rough silver electrodes was first discovered, the preparation of SERS-enhanced substrates and the optimization of their properties have never stopped, and the development of SERS substrates has gone through the following phases: (1) the preparation of SERS substrates with inhomogeneous surface roughness by vacuum deposition or electrochemical redox cycling [3,5,6]; (2) the preparation of micron-sized and broadly dispersed sol–gel particles by laser fusion or wet chemical synthesis [7,8,9,10,11,12,13]; (3) the preparation of micron-sized and broadly dispersed sol–gel particles by laser fusion or wet chemical synthesis [14,15,16]; and (4) the preparation of SERS substrates by laser fusion or wet chemical synthesis [17,18]. In particular, with the rapid development of nanoscience and technology in the past three decades, the preparation of SERS substrates has become more and more sophisticated, and the control of substrate structure has become more and more mature, thus greatly facilitating the development of SERS in the fields of chemical analysis and biomedicine.

Nanomaterials have a very important impact on the present and development of society. They are one of the three pillar sciences of the 21st century, which are the most active in the field of new materials and are considered to be the closest to practical applications [19,20,21]. Nanomaterials are broadly defined as materials with at least one dimension and referred to as substances with a size of 1 nm–100 nm in at least one dimension, which is a mesoscopic system between micro and macro. These materials have many properties that are very different from those of traditional macroscopic materials, such as (a) dielectric domain-limiting effect, which refers to the fact that charge-carrying power lines in semiconductor nanoparticles wrapped in dielectric constants smaller than those of bare nanoparticles are more prone to pass through the wrapping film, thus changing their optical properties. When the difference in dielectric constant is large, this effect is more obvious, and the absorption spectrum is larger [22,23]; (b) quantum size effect, which indicates that when the size of a particle is as small as a certain value, the phenomenon of changing the electronic energy level near its Fermi energy level leads to a huge difference between the optical, magnetic, thermal, acoustic, electric and superconducting properties of the nanoparticles and their macroscopic properties [24,25]; (c) surface effect, which is characterized by a high surface energy and a high proportion of surface atoms, thus make the nanoparticles very unstable and prone to the phenomenon of aggregation and binding, which affects the changes of atomic configuration and electronic energy spectrum. These properties make nanomaterials have significant SERS effects and give them a broad prospect for application in SERS substrates [26,27,28,29,30,31,32].

Great SERS substrates are characterized by (1) higher SERS effect, providing detection sensitivity [33]; (2) high SERS signal reproducibility, improving detection reliability [34]; (3) SERS signal homogeneity, further improving detection reliability [35]; (4) high stability, prolonging the service life of the substrate [36]; and (5) practical and simple preparation process, low preparation cost, as well as low application cost to increase practical applicability. However, it is still difficult to prepare SERS substrates that satisfy these demands simultaneously [37]. Currently, common materials with SERS effect include precious metals (silver, gold, copper, etc.), alkali metals (lithium, sodium, potassium, etc.), transition metals (iron, cobalt, nickel, etc.), platinum group metals (platinum, ruthenium, rhodium, etc.) and graphene. Among them, the most researched and best-performing SERS substrates are those based on metal materials. Herein, several representative metal based-SERS substrates have been introduced below.

## 2. Preparation of SERS Substrates

### 2.1. SERS Substrates Based on the Disordered Nanoparticle Aggregates Structures

#### 2.1.1. Metal-Based Nanoparticles, Nanowires, and Nanostar SERS Substrates

Some metal conductors are used as raw materials for SERS substrate due to their superb surface plasmon effect [38,39,40,41,42,43]. Among them, gold and silver nanoparticles are the most frequently used raw materials. Wet chemical synthesis is a widely used method for preparing gold and silver nanoparticles by redox reactions on metal salts in the presence of ligands. In 1982, Lee et al. [44] proposed the reduction of silver nitrate with sodium citrate, which can finally obtain silver nanoparticles with a particle size of about 60 nm. The particle size of the metal nanoparticles is closely related to the strength of the local electric field on their surface, thus playing an important role in the SERS effect. Subsequently, Frens et al. [45] and Natan et al. [46] successfully adjusted the particle size by increasing or decreasing the amount of sodium citrate. As a result, they found that the local electric field strengths on the surface of nanoparticles with different particle sizes are different, and the strength of the SERS effect is different.

Silver nanoparticles could show a strong SERS effect. However, their surface is susceptible to oxidation, which greatly reduces their SERS effect. Therefore, attempts have been made to prepare gold and silver biocomponent nanoparticles to improve the antioxidant stability of these nanoparticles, thanks to the special electronic interactions between these two components. These attempts were also utilized to prepare substrates with a strong SERS effect [47,48]. It has been demonstrated that with a silver ring on the surface of gold nanoparticles, the Raman detection sensitivity of gold will be impressively improved [49,50]. However, bimetallic nanostructures are still relatively unstable in the air and would be easily oxidized and aggregated in solutions. Facing this challenge, researchers have proposed to grow another protective layer on the surface of metal nanoparticles using the core–shell structure approach to maintain the stability of metal nanoparticles in different environments. For example, Liz et al. [51] reported a SERS substrate with the use of plasma particles encapsulated in SiO_2_ shell layers for the first time. They investigated the SiO_2_ coating mechanisms and found that the SiO_2_ shell layers could protect the substrates from oxidation. Later, Nie et al. [52] and Natan et al. [53] used silane coupling agents and probe molecules to modify the SiO_2_ encapsulated plasma nanoparticles with core–shell structures. They also showed an active substrate, such as SERS labels. After that, Du et al. [54] optimized the experimental conditions and successfully prepared Ag@SiO_2_ core–shell SERS active substrates with probe molecules adsorbed on the surface of silver particles. Protected by the SiO_2_ shell layer, the SERS substrate is more stable and durable. With further research on SERS substrates, another class of shell-isolated nanoparticle SERS substrates has been developed [55]. Similarly, the gold nanoparticles were wrapped in an ultra-thin layer of alumina or SiO_2_ without any probe molecules. Results showed that these shell layers could completely isolate the gold nanoparticles from contacting with probe molecules and significantly enhance their stability and utility.

**Figure 1:**
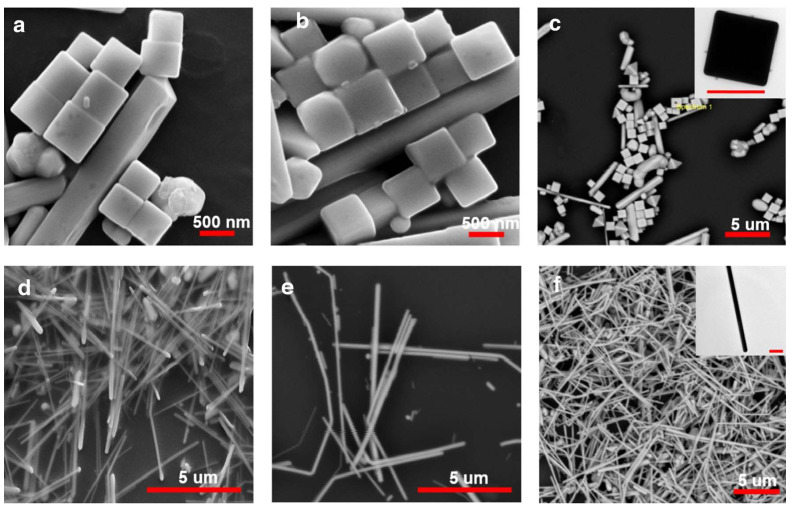
SEM images (**a**–**c**) silver nanocubes (**d**–**f**) silver nanowires SEM images (**a**–**c**) silver nanocubes (**d**–**f**) silver nanowires (the inset shows the TEM micrograph of corresponding nanocube and nanowire with a scale bar of 500 nm) [57]. Copyright © 2020, Springer.

Anisotropic metallic nanomaterials possess greater surface plasmon resonance wavelength tuning and electromagnetic “hotspots” attributed to their physical structures. Thus, these anisotropic metallic nanomaterials have stronger SERS effects. Among them, metallic nanowires have been frequently used in biomedical applications such as tumor cell labeling and bioimaging due to their crystalline structure, which could result in higher SERS effects (surface plasmon excitations would propagate on smooth metallic nanowires with a very low loss). Currently, polyalcoholization is the main method for preparing silver nanowires (micrometer in length and 30–60 nm in diameter). For example, Tao et al. [56] used this method to prepare tightly packed and ordered silver nanowires. In this study, they verified that the strong electromagnetic coupling between silver nanowires would excite a very strong localized electric field, resulting in an ultra-strong SERS effect. Besides, Kumar et al. [57] synthesized silver nanowires and silver nanocubes using polyols by modulating different synthesis parameters such as reaction molar ratio, temperature, and reaction time. The nanowires and nanocubes are shown in Figure 1. Both synthesized silver nanowires and nanocubes showed sharp structural features as well as different SERS activities. Furthermore, compared with the nanowires, the substrates of nanocubes exhibit higher SERS activity, mainly due to the “hot spots” generated by the sharp tips of the nanocubes as well as the formation of more dimeric structures between the nanogaps.

Star-shaped nanomaterials have also shown significant potential for applications ranging from SERS sensing and imaging to photothermal therapy and photoimmunotherapy for cancer treatment. The validity and reliability of star-shaped gold nanoparticles for biomedical applications depend largely on the stability and reproducibility of the physical, chemical, and optical properties of the nanoparticles, which in turn depend largely on the morphological characteristics of the star-shaped gold nanoparticles. For example, De et al. [58] prepared surfactant-free star-shaped gold–silver heterogeneous nanoparticles by optimizing a “bottom-up” synthesis method, using ascorbic acid as a capping agent, and adjusting the order of reagent additions and the ratio of gold ions to silver ions in the reaction solution, which ultimately led to the modulation of the number and length of spikes of star-shaped gold nanoparticles. The number of spikes and the length of spikes of the star-shaped gold nanoparticles were adjusted to further realize the high reproducibility of the morphology and optical characteristics of the star-shaped gold-silver heterogeneous nanoparticles from different synthesized batches. It was also found that the optical response of star-shaped gold nanoparticles with different numbers and lengths of spikes was different, which lays an important foundation for the preparation of SERS substrates of star-shaped gold–silver heterogeneous nanoparticles with optimal synthesized performance.

**Figure 2:**
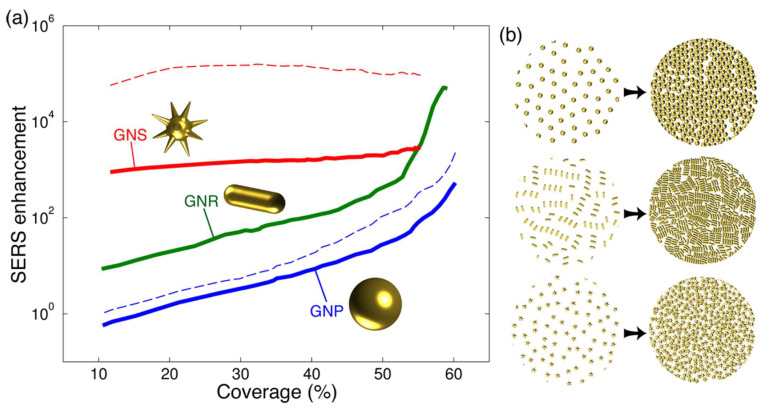
Density dependence of the SERS performance in nanoparticle monolayers. The particle coverage is defined as the fraction of area occupied by the projection of the metal along the normal plane (solid curves and dashed curves represent different incident wavelengths) [59] (**a**,**b**). Copyright © 2016, American Chemical Society.

The strength of the substrate SERS effect depends on the morphology and nature of the nanoparticles, and many experiments and computational simulations have demonstrated that plasma particles with sharp protruding structures always show stronger SERS activity. However, the plasma particle geometries in numerical simulations are generally relatively simple when it is more important to take into account the large-scale aggregation effect of plasma particles in practical applications. For example, Solis et al. [59] used state-of-the-art electromagnetic computational simulation techniques to study the SERS enhancement factor (EF) values of hundreds of gold nanoparticles with different morphologies in a random aggregation state, which is shown in Figure 2a,b. The conclusions unexpectedly showed that nanoparticles providing large EFs in the single-particle case, e.g., nanostars, do not necessarily enhance their EFs when they are densely packed. On the contrary, simpler morphologies (e.g., spherical or rod-shaped nanoparticles) could significantly enhance their SERS EFs when they are densely packed. These can be explained by the fact that the surface density of simpler nanoparticles is closer to the full coverage when they are densely arranged, thus generating a larger number of “hot spots” and a higher EF.

#### 2.1.2. Preparation of SERS Substrates with Surface-Immobilized Metal Nanoparticles

**Figure 3:**
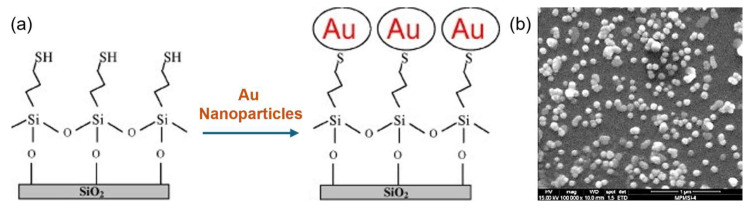
(**a**) Schematic representation of the SERS substrate fabrication procedure. (**b**) SEM images of Au-colloidal films deposited immediately [61]. Copyright © 2009, American Chemical Society.

Nanoparticle suspensions are dynamic systems that are pretty unstable and prone to aggregate to different degrees over time. When nanoparticle suspensions are used as SERS substrates, the stability and reproducibility of the SERS signal are very low, which severely limits its applications. To address the instability of the suspension SERS substrate, as well as to simultaneously utilize the aggregation of nanoparticles to further enhance the SERS effect, an effective approach is to immobilize the nanoparticles on some solid substrate. For example, the method of chemically immobilizing nanoparticles on the surface of a solid substrate is known as the “chemical tethering” method [60]. As shown in Figure 3, the surface of a silicon wafer is silanized with (3-mercaptopropyl) trimethoxysilane to attach a chemical chain with a sulfhydryl group at the end. When the treated wafer is immersed in a suspension of silver and gold nanoparticles prepared with sodium triacetate, the sulfhydryl groups will attach to the surface of the wafer, tethering the nanoparticles to the surface of the wafer. It can be observed that the nanoparticles are uniformly and densely distributed on the wafer surface [61].

**Figure 4:**
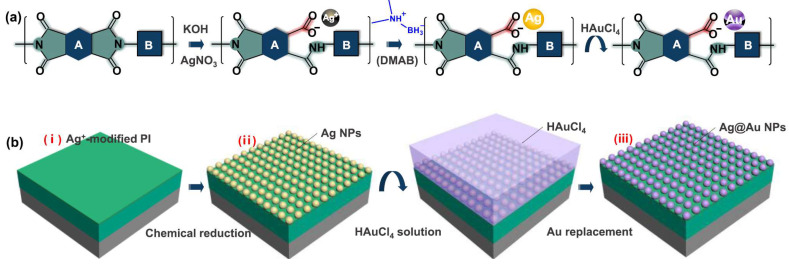
Schematic illustration of (**a**) nanoparticle growth on the colorless polyimide molecular chain and (**b**) corresponding flexible SERS sensor fabrication: (**i**) modification of PI; (**ii**) growth of Ag nanoparticles; (**iii**) growth of Ag@Au nanoparticles [62]. Copyright © 2020, American Chemical Society.

Furthermore, Liu et al. [62] reported a method to grow nanoparticles in situ using polymers as templates, as shown in Figure 4. First, the polyimide film was immersed in an aqueous solution of potassium hydroxide to hydrolyze the imide ring of the polymer chain to release the carboxyl group. Then, the polyimide film is immersed in silver nitrate solution, where charge attraction and coordination between the negatively charged carboxyl group and silver ions occurs, restricting the silver ions to the surface of the film. As a result, silver nanoparticles were successfully synthesized in situ by redox reaction. The prepared film continued to be transferred to chloroauric acid solution for a gold–silver substitution reaction. Finally, large-area gold-coated silver nanoparticle structures with uniform distribution and dense arrangement were synthesized on the surface of polyimide films. There is also a strong bonding force between nanoparticles and the polyimide films. Results showed that the polyimide-gold-coated silver nanoparticle substrate has high SERS activity and great potential for practical applications, which is characterized by high signal uniformity and high detection sensitivity.

**Figure 5:**
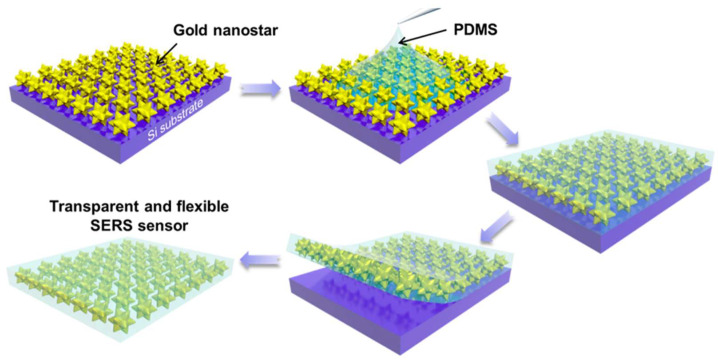
Schematic illustration showing the fabrication process of a flexible SERS sensor with gold nanostar arrays. Self-assembled gold nanostar arrays are transferred from silicon substrate into polydimethylsiloxane (PDMS) [63]. Copyright © 2017, Royal Society of Chemistry.

A flexible star-shaped gold nanoparticle SERS substrate was prepared by blending star-shaped gold nanoparticles into a polydimethylsiloxane (PDMS) film, as shown in Figure 5 [63]. The flexible substrate could contact the surface of non-planar objects, which improves the efficiency of Raman detection sampling and expands its practical application. The optical transparency of the PDMS film allows light to penetrate and contact the surface of the object, thus enabling the highly sensitive detection of probe molecules adsorbed on the surface of any metal or dielectric object. As a result, the flexible star-shaped gold nanoparticle substrate successfully detected the Raman signals of benzenethiol molecules at a concentration of 10^−8^ M, with an EF as high as 1.9 × 10^8^. Furthermore, the star-shaped gold nanoparticles were monolayered inside the PDMS film and maintained a relatively good state and high SERS sensitivity after 100 cycles of mechanical deformations, such as tensile stretching, bending, and twisting. In addition, it was also found that the Raman signals of the probe molecules attached to the non-SERS “hot spots” were significantly enhanced by the strong electric field enhancement at the tips of the star-shaped gold nanoparticles. Moreover, Wang et al. [64] proposed a facile method to fabricate a flexible SERS substrate by self-assembling Au nanoparticles on PDMS nanowrinkles. The nanowrinkled PDMS substrate is prepared through a tunable nano-wrinkle generation process, which enhances surface roughness and subsequently improves SERS signal enhancement. Compared to a SERS substrate prepared on flat PDMS, the nano-wrinkled SERS substrate demonstrates a significantly higher EF of approximately 4.4 times. This enhancement is attributed to the increased surface roughness and formation of “hot spots” between Au nanoparticles on the nanowrinkled PDMS surface.

### 2.2. SERS Substrates Based on Ordered Nanoparticle Aggregate Structures

#### 2.2.1. Preparation of SERS Substrates by Nanosphere Etching Technique

**Figure 6:**
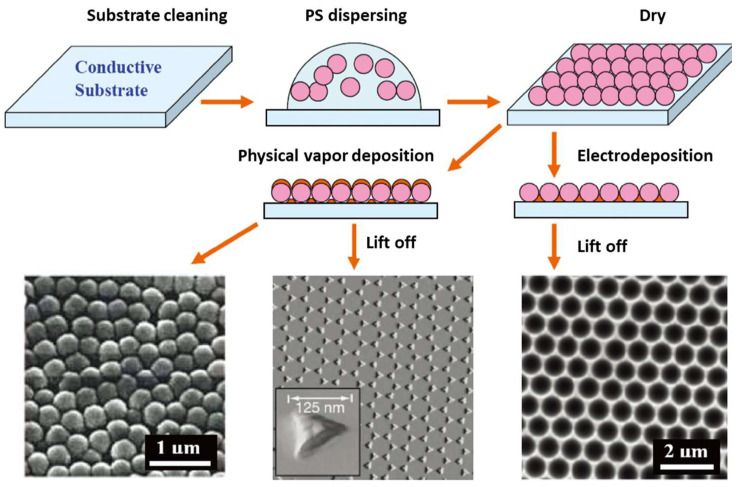
Template methods using nanosphere lithography to fabricate ordered nanostructured SERS substrates [66,67,68]. Copyright © 2007, Royal Society of Chemistry.

The nanosphere etching technique is used to prepare microscopic nano-arrays with highly ordered structures and is very cost-effective. As a technique, monodisperse silica or polystyrene nanospheres are deposited on the surface of a conductive substrate (e.g., metal-coated glass or indium tin oxide) to form a self-assembled ordered array, i.e., a microsphere array template. The SERS substrate is formed by plating a thin metal film on the surface of the array template and removing the self-assembled arrays. The first SERS substrate was prepared using nanosphere etching [65]. One or more layers of self-assembled microsphere arrays on the surface of a solid substrate were coated with a metal film on the surface of the microsphere arrays by deposition. Then, nanopore arrays were obtained by electrochemical deposition, while nano triangular arrays could be prepared by physical vacuum deposition. The deposition angle can be adjusted to obtain different shapes of nano-arrays, while the deposition thickness can be changed to obtain nanostructures with different sizes and spacing, thus obtaining SERS substrates with different structures and effects [66,67,68]. The use of nanosphere etching to prepare ordered arrays of various patterned nanostructures has greatly promoted the study of controllably prepared patterned nanostructures, as shown in Figure 6. The difficulty in preparing SERS substrates using the nanosphere etching technique lies in the preparation of microsphere arrays, which require precise and stringent experimental conditions and experimental manipulations. If these experimental conditions are satisfied, micrometer-scale or even millimeter-scale microsphere arrays can be prepared. Nevertheless, microsphere arrays with diameters smaller than 200 nm are still difficult to prepare.

#### 2.2.2. Preparation of SERS Substrates by Electron Beam Etching and Photolithography

**Figure 7:**
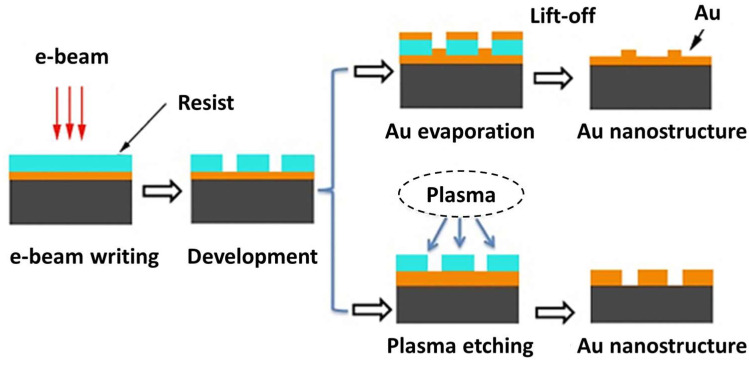
Schematic diagram of two processes for preparing SERS substrates by electron beam etching technique [69]. Copyright © 2012, IOP Publishing, Ltd.

Electron beam etching, a nanofabrication technique for preparing ordered arrays, has also long been utilized to prepare SERS substrates [69,70,71,72,73,74,75,76,77]. A typical process for the preparation of nanostructured SERS substrates by electron beam lithography is divided into two main steps. The first step is the electron beam drawing stage, which uses a focused electron beam to draw a customized pattern on the surface of a gold-plated wafer covered with a resist (electron-sensitive film). The second step is the “development” phase, where the electron beam is used to alter the solubility of the resist in the solution, removing exposed or unexposed areas of the resist by dissolution, leaving the remaining undissolved portion to be used as a template for the preparation of the SERS substrate. As shown in Figure 7, one preparation method involves depositing gold onto the surface of the template and then dissolving away the remaining resist to obtain a patterned nanostructured SERS substrate that conforms to the shape of the template. Another method involves a plasma etching technique in which a focused ion beam is directly irradiated onto the template so that the gold outside the resist-exposed region is removed by etching, and the gold in the region masked by the resist is retained because it is not irradiated, thus producing another type of SERS substrate. Although the prepared substrate has good Raman enhancement uniformity, the difficulty of the electronic etching technique lies in the etching of nanopatterns, which seriously limits its application in the preparation of SERS substrates.

Holographic lithography was also used to prepare SERS substrates with ordered gold arrays. A smooth silver layer was plated on the surface of the template by pulsed electrodeposition to prepare an array with an ultra-narrow nanogap, which has a highly ordered structure and a high SERS effect [78]. Moreover, Yu et al. [79] developed a reusable and consistent Ag-based SERS substrate using the Scanning Probe Lithography technique. This method involves using a diamond tip to machine the Ag surface and create micro/nanostructures that enhance the Raman signal. The method showed potential for application on metals like Au, Ag, and Cu, with Ag showing the best enhancement effect. The article contributes to the development of more efficient and cost-effective SERS substrates for advanced detection techniques. Compared with other SERS substrate preparation techniques, the main advantage of electron beam etching is the ability to draw customized patterns with a resolution of 10 nm, which is very important for the preparation of SERS substrates. This advantage is important for SERS substrate preparation because the localized surface plasmonic resonance of the SERS effect is highly dependent on the size, shape, and stacking of the nanostructures. However, nanostructures with spacing less than 10 nm are difficult to prepare by electron beam etching since nanolithography requires a very high cost, making it unsuitable for large-scale practical applications.

#### 2.2.3. Preparation of SERS Substrates by Polymer Template-Induced Self-Assembly

With the development of polymer research, the use of polymers as templates for the preparation of patterned nanostructures has been increasing. This includes the use of polymers for the preparation of nanostructured SERS substrates. For example, Yin et al. [80] reported a template-induced technique and prepared micrometer-sized arrays of bimetallic honeycomb structures consisting of self-assembled nanoparticles. First, porous patterned polystyrene films were prepared, and then the films were transferred to the surface of a silicon wafer whose surface was modified with sulfhydryl groups to prepare for the deposition of Pt in the next step. Then, Pt was sprayed on the surface of this wafer, and the area covered by the polystyrene film was free of Pt. Only the exposed wafer area in the circular holes of the film was sprayed with Pt so that a patterned Pt circular table array could be obtained. The silicon wafers were then immersed in a gold chlorate solution while ascorbic acid was added for the redox reaction. The generated gold nanoparticles were then surrounded around the edges of the Pt roundtables under the modification of polyvinylpyrrolidone, resulting in the formation of a bimetallic honeycomb-structured ordered array. This bimetallic ordered array showed a significant SERS effect. As a result, the array could detect aminothiophenol molecules at 10^−9^ M with excellent signal reproducibility. The relative standard deviation values of Raman intensities in different regions were as low as 3.3%. The synthesis method and pathway of this substrate do not involve complex chemical and physical processes, which developed a new route for the preparation of facile controlled self-assembled nanostructures.

In the realm of SERS technology, numerous innovations have been made to enhance detection capabilities, create versatile substrates, and expand the range of applicable materials. Here, we integrate a series of studies that showcase the diversity and advancement in SERS applications. For example, Li et al. [81] presented a novel SERS fiber probe fabricated through a combination of femtosecond laser ablation and photoreduced deposition of silver nanoparticles on a polymethylmethacrylate (PMMA) polymer fiber. The study developed a flexible and sensitive SERS probe for biochemical analysis applications and concluded by summarizing the fabrication process, optimizing laser pulse energy and scan surface period, and demonstrating the SERS probe’s sensitivity. The proposed PMMA-based SERS fiber probe offers a promising alternative for in situ measurement of SERS signals and further fabrication of SERS substrates on other materials such as silica fibers or silicon wafers. The flexibility and ease of fabrication of the polymer fiber make it a suitable candidate for biochemical analysis applications where softer and less fragile SERS probes are preferred. Schiller et al. [82] demonstrate the preparation of biotinylated, self-assembled polymer-stabilized gold nanoparticle hybrids encoded with an SERS active compound and their potential use in bioassays. The polymers used for nanoparticle stabilization were carefully designed and synthesized by the RAFT polymerization process, and the functionalized biotin moieties were attached to the hybrid nanoparticles via Cu-catalyzed azide-alkyne cycloaddition. The study includes the requisites that constitute a bioassay, showcasing the potential of polymer-coated hybrid nanoparticles for this purpose. This work contributes to the advancement of SERS-based bioassays by providing a novel and promising approach for the detection and identification of analytes. Strozyk et al. [83] reviewed various strategies for the synthesis and functionalization of these composite colloids, including encapsulation of Au nanoparticles within polymeric shells. They discussed the encapsulation process, which is often based on hydrophobic interactions between a polymeric ligand shell and a suitable block-copolymer, resulting in stable composite colloids in aqueous solutions. These colloids exhibit unique optical and structural features that make them suitable for SERS sensing, along with other applications such as drug delivery, imaging, and catalysis. Furthermore, the article introduces the concept of smart polymer composites, which change their structure in response to external stimuli or environmental changes. This includes thermoresponsive polymers like poly(*N*-isopropylacrylamide), whose lower critical solution temperature can be finely tuned, making them attractive for various applications. The authors demonstrate the use of these smart polymers in the encapsulation of Au nanoparticles, creating hybrid colloidal materials that can be used as substrates for generalized SERS detection in aqueous solutions. Overall, the article offers valuable insight into the recent advances in the field of composite polymer colloids for SERS-based applications. Their work not only enhances our understanding of the encapsulation and functionalization of Au nanoparticles within polymeric shells but also opens up new opportunities for the development of novel SERS sensors and other related technologies.

Chen et al. [84] developed a SERS gas sensor matrix with high design flexibility, utilizing multiple polymer films to enhance gas recognition capabilities. The study demonstrated the successful discrimination of phenethyl alcohol, acetophenone, and anethole through the application of a principal component analysis algorithm on the response matrix constructed by the combination of gas responses obtained from one-layer and two-layer film-coated sensors. This innovative approach provides a new perspective for SERS sensing methods in the recognition of gases with similar molecular structures. Fularz et al. [85] have demonstrated that cellulose nanofiber-based substrates can serve as an effective metal-free platform for SERS of porphyrin-type molecules. Their study revealed a significant enhancement in SERS signal, highlighting the potential of these renewable and sustainable organic polymers in molecular detection and sensing applications. Chen et al. [86] creatively employed poly(3,4-ethylenedioxythiophene):poly(styrenesulfonate), a p-conjugated conducting polymer, as a metal-free SERS substrate to investigate the carrier dynamics. The authors demonstrate that poly(3,4-ethylenedioxythiophene):poly(styrenesulfonate) exhibits excellent SERS activity, with an EF of up to 2.26 × 10^3^ for methylene blue as a probe molecule. Furthermore, they evaluate the SERS performance of poly(3,4-ethylenedioxythiophene):poly(styrenesulfonate)/methylene blue under various bias voltage conditions, revealing that the intensity and shift of the SERS peaks are correlated with the carrier density and charge transfer process. The work not only provides an important theoretical basis for the establishment of chemical enhancement models in SERS but also offers a new perspective for the exploration of photoelectric devices. Nguyen et al. [87] developed a novel SERS substrate by synthesizing a hybrid structure consisting of Au nanorods coated with a molecularly imprinted polymer. This Au nanorod@molecularly imprinted polymer substrate demonstrated a significant enhancement in SERS signals, particularly for rhodamine B, offering a promising approach for sensitive and specific SERS-based detection of dyes in various samples. Wei et al. [88] synthesized polymer-grafted plasmonic metal nanoparticles that show a significant enhancement in SERS activity under acidic conditions. The authors revealed that the pH-responsive polymers, such as poly(acrylic acid) and poly(allylamine hydrochloride), can induce aggregation of the nanoparticles upon changes in solvent quality, leading to the formation of hot spots and a subsequent increase in SERS signal. This innovative approach allows for SERS detection in solutions without the need for sample pre-concentration, offering a promising avenue for the development of sensitive and dynamic sensing platforms. Lin et al. [89] presented an innovative low-power density reflective Raman system designed to address the challenge of high heat concentration in polymer substrates during SERS measurements. Their study demonstrated that the reflective Raman system not only reduced the input power density but also maintained high collection efficiency, thus minimizing potential damage to sensitive analytes and SERS substrates. This advancement could significantly benefit the analysis of thermally labile samples, such as certain polymers and biomaterials.

**Figure 8:**
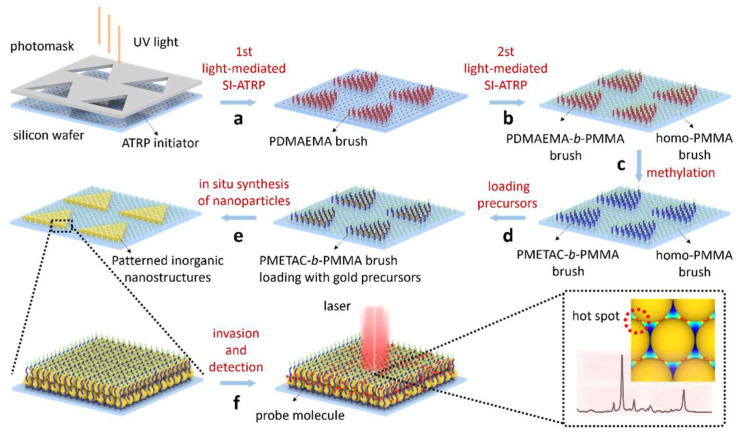
Schematic illustration of the in situ synthesis for patterned Au nanostructures with tunable shape and size templated for SERS applications [93]. Copyright © 2022, Tsinghua University Press.

Large-area arrays of plasma clusters arranged in an orderly fashion were prepared using square arrays of PDMS containing submicron porous dimensions as templates [90,91,92]. The porous size of the PDMS molds determines the diameter and lattice parameters of the gold nanostructures, while the gold nanoparticle colloidal concentration determines the number of layers and the self-assembly quality of the plasma clusters. In addition, the correlation of plasmon resonance and the corresponding SERS effect between gold nanoparticle clusters prepared with different porous sizes of molds were investigated. Significant differences in the SERS effect of the plasma clusters with different geometries were found. In another study, a robust strategy to generate tailor-made and efficient SERS platforms by using patterned amphiphilic block copolymer brushes for assembling Au nanoparticles into highly uniformly distributed and densely packed plasmonic nanoparticles was developed (Figure 8) [93]. To achieve this, patterned polymethacryloyloxyethyl trimethylammonium chloride-block-polymethyl methacrylate brushes are grown on a substrate using the light-mediated surface-initiated atom transfer radical polymerization technique assisted by photomasks. The inner hydrophilic domains are utilized as nanoreactors for the formation of Au nanoparticle assemblies, while the outer polymethyl methacrylate blocks serve as confinement. Following the polymer brushes template-assisted in situ nucleation and growth approach, patterned quasi-two-dimensional (2D) Au nanostructures with controllable size and shape can be feasibly fabricated under ambient conditions.

These studies collectively represent a giant leap in SERS technology, offering innovative solutions for substrate fabrication, signal enhancement, and sensing applications in diverse fields, including biochemistry, environmental monitoring, and material science.

#### 2.2.4. SERS Substrates Based on Metal-Organic Frameworks (MOFs)

Metal-organic frameworks (MOFs), which are crystalline porous structures made from metal ions and organic linkers, have seen widespread use in various applications such as catalysis, energy storage, and separation processes [94,95,96,97,98]. These materials are prized for their low density, high porosity, substantial specific surface area, high crystallinity, tuneable pore dimensions, and a variety of topological configurations. Given these characteristics, MOFs are well-suited to be utilized as substrates for SERS applications, addressing several challenges: (1) their expansive surface area offers numerous sites for capturing target analytes; (2) the porous nature and crystallinity of MOFs allow for selective permeability; (3) the MOF shell can shield nanoparticles from oxidative and corrosive damage, thereby enhancing the stability of the SERS substrate; (4) the facile tailoring of MOF structures makes it possible to design SERS substrates for the detection of specific molecules [99,100,101,102,103]. There have been recent comprehensive reviews on the preparation and characterization of MOF-based SERS substrates, although the underlying design principles for such substrates have not been fully elucidated.

**Figure 9:**
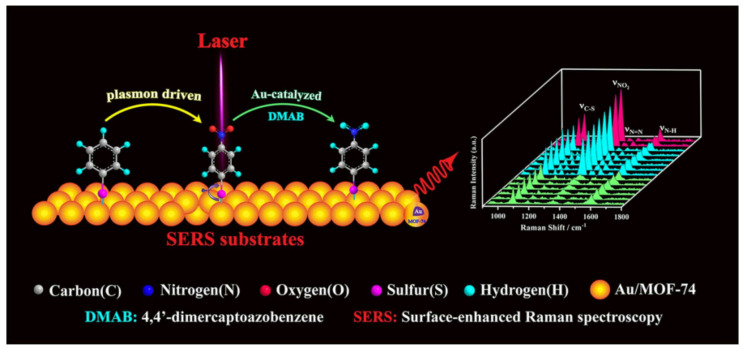
Schematic presentation of MOF-integrated SERS substrate consisting of gold core and MOF-74 shell, which was used for in situ SERS monitoring of model reaction [105]. Copyright © 2019, Springer.

For example, Qiao et al. [104] designed an elaborate SERS substrate by coating gold superparticles with a ZIF-8 layer to detect lung cancer biomarkers. The coated ZIF-8 layer could not only enhance the adsorption of gas molecules but also slow down the flow rate of gas biomarkers while suppressing the exponential attenuation of the electromagnetic field around the superparticles. The detection limit of gaseous aldehydes is 10 ppb, showing great potential for in vitro diagnosis of early lung cancer. Zhang’s group designed Au/MOF-74 composite NPs with a core–shell structure through a one-step synthesis method (Figure 9) [105]. It not only exhibited excellent SERS performance but also had high sensitivity and stability in quantitative analysis. More importantly, the Au/MOF-74 nanocomposite could be used for in situ SERS monitoring of the nitration of aromatic rings without adding a conventional acid catalyst. It is speculated that the MOF shell could enrich the reactive molecules and facilitate an efficient hot electron transfer process. Zhang et al. [106] articulated a methodical synthesis process involving the thermal treatment of Ag-MOF, which results in the formation of Ag nanoparticles dispersed on a carbonized MOF framework. This process ensures a uniform distribution of Ag nanoparticles with a precise spacing distance of approximately 7 nm, which is pivotal for maximizing SERS activity. The material’s efficacy is demonstrated through its application in detecting organic compounds such as methylene blue, malachite green, and crystal violet with remarkable sensitivity and specificity, showcasing a detection limit as low as 10^−8^ M. They also provided a comprehensive theoretical analysis and experimental validation of the SERS activity of their engineered material. Simulations of the electromagnetic field distribution further substantiate the material’s potential for SERS applications. The study concludes with the prospect of this strategy guiding the design of future SERS active materials with enhanced reproducibility and activity. This work offered a seminal reference to the development of SERS-based sensors for the detection of water-soluble organic compounds, and their findings underscored the potential of MOF-engineered materials to transform analytical chemistry and environmental monitoring. Ge et al. [107] developed three-dimensional (3D) Au/MOF-808 (Zr) composite nanostructures using a self-assembly method, which was found to be highly effective as SERS substrates for the detection of thiram, a common organosulfur pesticide. The study demonstrated that the optimal SERS substrates could detect thiram concentrations down to 10^−10^ M with high sensitivity and good adsorption, homogeneity, and reproducibility, indicating their potential for sensitive detection of pesticide residues in food and environmental samples.

In a recent development within the field of environmental analysis, Xu et al. [108] have engineered a novel composite material for the sensitive detection of thiram, a prevalent pesticide. Their study introduced a layered structure of filter paper–silver nanoparticle-ZIF-8 (FP/Ag/ZIF-8) designed to enhance the SERS detection of pesticide residues. This substrate demonstrates a heightened adsorption capacity and an optimized SERS response attributed to the thick zeolitic imidazolate framework (ZIF-8) coating. The research detailed a meticulous preparation process, resulting in a substrate that not only provides a low detection limit for 4-aminothiophenol, a pesticide intermediate but also exhibits versatility in capturing thiram from various real-world samples. This includes applications in lake water, peach juice, and apple peel through soaking, filtration, or swabbing operations. The substrate’s performance highlights the potential of layered plasmonic particle/MOF hybrids for toxicant analysis in environmental and food safety domains. The author further validated the substrate’s reproducibility, stability, and size-selective response for thiram detection. Their work contributes significantly to the advancement of SERS-based detection techniques, offering a practical and efficient tool for monitoring pesticide residues in diverse matrices. Chen et al. [109] described the process of dispersing ZIF-8 nanocrystals on a substrate, followed by the application of an Ag layer to create plasmonic nanogaps as small as 10 nm. These nanogaps, situated between the Ag nanocap atop the ZIF-8 nanocrystals and the surrounding Ag layer, leverage plasmonic coupling effects to generate a substantially enhanced electromagnetic field. This enhancement is further supported by numerical simulations and the pre-concentration capabilities of the porous ZIF-8 nanoparticles, which can enrich analytes within the nanogaps. The study presented compelling results, demonstrating a SERS EF of up to 2.84 × 10^7^, the ability to detect 4-aminothiophenol at concentrations as low as 10^−9^ M, and a relative standard deviation of approximately 6.99%. These findings underscore the potential of the Ag/ZIF-8 substrate as a robust SERS platform for molecular detection, offering a promising strategy for the design of MOF-based nanostructure substrates. A recent study by Zhao et al. [110] presents an innovative approach utilizing Au/Fe_3_O_4_/MOF-867 nanoparticles as a SERS substrate for the rapid determination of thiram in lake water. The study details a methodical synthesis of the Au/Fe_3_O_4_/MOF-867 composite, which integrates the magnetic properties of Fe_3_O_4_, the porosity of MOF-867, and the plasmonic characteristics of gold nanoparticles to create an efficient SERS substrate. This work meticulously optimized the composition of the substrate to achieve a remarkable limit of detection of 3.8 × 10^−10^ M for thiram, underscoring the high sensitivity of their method. The study demonstrates the substrate’s potential for environmental analysis, with implications for monitoring and managing pesticide residues in aquatic ecosystems. The authors contribute to the field by providing a detailed characterization of the Au/Fe_3_O_4_/MOF-867 composite, including scanning electron microscopy, transmission electron microscopy, energy dispersive spectroscopy, X-ray diffraction, and magnetic property measurements.

Over recent years, the burgeoning field of MOF-based SERS substrates has garnered significant attention, fueled by advancements in MOF materials and a growing need for highly sensitive detection methods. MOFs and their composites have demonstrated substantial potential in the realm of SERS [111,112,113,114,115,116,117,118]. They offer a distinct edge over conventional SERS substrates by providing a range of benefits, including heightened sensitivity, enhanced stability, improved selectivity, and significant customizability, allowing for the straightforward development of MOF-based materials [119,120,121,122,123,124,125,126]. While the synthesis of MOF-based SERS substrates is generally more straightforward than that of traditional counterparts, there are concerns regarding the toxicity of certain key organic ligands and solvents used in the process. There is a future demand for the development of environmentally friendly preparation techniques. Additionally, for the commercialization of MOF-based SERS substrates, there is a necessity to achieve large-scale production methods.

#### 2.2.5. Other Types of SERS Substrates

A common method of testing SERS substrates is to use an optical microscope to focus the incident light into a very small point and then irradiate it on the surface of the sample for testing. For samples that require remote or in situ measurements, the focus of the laser is very difficult. To perform in vivo as well as in situ SERS testing, Vo-Donh et al. [127] coated silver on the surface of alumina nanospheres and then integrated the nanospheres into a fiber optic tip to prepare a SERS sensor at the fiber optic tip. The size of the SERS substrate can be modulated by changing the size of the fiber tip to achieve in situ SERS detection on the nanoscale. Moreover, the use of nanoimprinting for the preparation of nanostructures is more cost-effective and efficient than photolithography. Photolithography has been utilized to make patterned molds on silicon wafers. Then, the molds are imprinted on the polymer surface of the substrate. After curing the polymer, the polymer templates with ordered patterned arrays were obtained by tearing them off from the templates. Finally, the metal nanoparticles were deposited on the surface of the polymer templates, and the nanoparticles self-assembled within the templates’ patterns to form the corresponding patterned nanoarrays. Furthermore, Yang et al. [128] prepared large-area 2D patterned nanoporous gold arrays using nanoimprinting techniques. The mesh structure inside the nanoporous gold is capable of generating a strong surface plasmon resonance effect, which leads to a strong SERS effect. In this work, porous nanoporous gold films with a strong SERS effect were processed into ordered nano-arrays, which further enhanced their SERS effect. The EF for benzene thiol molecules in the test was up to 10^7^ M. As a result, nanoimprinting the technique is more economically viable than photolithography. However, this technique is also limited by the precision of preparation, while it is difficult to use for the preparation of nanopatterned structures with a pitch of less than 10 nm. Moreover, Wang et al. [129] successfully prepared a SERS substrate with high-density hot spots on the inner wall of a capillary, which would enhance the SERS properties and make the substrate suitable for practical applications and sensitive detection of trace amounts. The substrate provided a convenient sampling method and showed excellent uniformity and reproducibility in detecting R6G and goat serum. Therefore, the work highlights the potential of the capillary SERS substrate as a promising and rapid in situ detection tool for various practical applications.

Various methods mentioned above for preparing SERS substrates require a lot of effort and cost. Therefore, attention has been focused on some materials in nature that could be used to prepare nanostructures. Cell walls of some marine microorganisms in nature possess an ordered photonic structural ordering, such as diatoms. Since diatoms are very abundant in the oceans, many diatom cell walls have been widely used in practical applications, mainly as drug carriers, luminescent materials, and solar cells [130,131]. For example, Wang et al. [132] used a diatom cell wall as a template and prepared SERS substrates by self-assembly of Ag nanoparticles on the cell wall surface. The substrate has a comparable SERS effect with better economic benefits and a wide range of application prospects. Moreover, natural products offer several advantages as substrates for SERS. In conclusion, natural products are advantageous SERS substrates due to their biocompatibility, renewability, cost-effectiveness, and eco-friendliness. Natural products have diverse structures that enhance SERS signals, and their functional groups can also improve sensitivity and selectivity. In addition, they are non-toxic and can be functionalized, thus offering a wide range of applications.

## 3. Conclusions and Outlook

With the continuous development of nanomaterials preparation technology and the continuous improvement of signal detection systems, SERS detection technology has been developed and played an indispensable role in many fields, with good prospects for development and broad application space. SERS stands as an exceptional precision analytical tool, distinguished by its unparalleled sensitivity and specificity in molecular detection. Its interdisciplinary utility and paramount importance are manifested in domains ranging from environmental conservation to forensic analysis. Within the realm of environmental monitoring, SERS technology emerges as a potent instrument for the precise identification of heavy metals, pesticides, and other detrimental pollutants, underpinning robust strategies for ecological preservation.

In forensic science, it assumes a pivotal role in uncovering trace yet crucial evidence that is indispensable for case investigations and solving. Moreover, SERS plays a fundamental part in biomedical research, facilitating precise disease diagnosis and biomarker discovery by virtue of its capability to detect biological entities. In the realm of food safety, it safeguards consumer well-being by identifying adulterants, while in pharmaceutical applications, it enables the accurate identification and quantitative analysis of drug active ingredients. Additionally, SERS contributes significantly to materials science by unraveling vibration modes and surface properties, thereby accelerating the development and performance evaluation of novel materials. In cultural heritage conservation, it analyzes pigments and inks to authenticate artifacts, while in security contexts, its ability to detect chemical threats fortifies security protocols. A pivotal advantage of SERS lies in its non-destructive and real-time monitoring prowess, indispensable for applications requiring continual assessment or sample preservation. In biomedical pursuits, this feature guarantees diagnostic precision and facilitates the comprehensive tracking of disease progression and treatment outcomes.

The fusion of nanotechnology with SERS has catalyzed profound advancements. By amplifying Raman signals from molecules adsorbed on metallic nanostructures, SERS offers unprecedented insights into nanoscale environments and interactions, spurring breakthroughs across various affiliated domains. Furthermore, the flexibility in substrate design and preparation techniques enables SERS to achieve remarkable selectivity and sensitivity in specific molecular detection. Remarkably, SERS also showcases robust integration capabilities, facilitating synergies with other analytical modalities like mass spectrometry and fluorescence spectroscopy, thereby augmenting its analytical efficacy and expanding its application horizon. Specifically, SERS-mass spectrometry offers comprehensive molecular characterization and structural insights, while SERS-FRET enables high-resolution monitoring of biomolecular interactions. As SERS equipment becomes increasingly accessible and affordable, coupled with advancements in data processing technologies, this advanced analytical instrument is democratizing advanced analysis, fostering scientific innovation and interdisciplinary collaboration, and catalyzing novel solutions to global challenges.

In conclusion, SERS, as a continuously progressing and transformative technology, has firmly established itself as an invaluable asset for scientific research and industrial applications, with its application frontiers continually expanding. With the maturing and popularization of this technology, SERS promises to exert an even more profound influence in domains such as biomedicine and environmental monitoring, ushering in a new era of innovation and progress.

## Data Availability

Data are contained within the article.
